# Impact of the COVID-19 pandemic on the education and procedural volume of fellows in critical care medicine – a cross-sectional survey

**DOI:** 10.1186/s12909-023-04358-2

**Published:** 2023-05-24

**Authors:** Orlando Garner, Kanta Velamuri, Kristen Staggers, Andrea Barbara Braun

**Affiliations:** 1 Department of Medicine, Division of Pulmonary, Critical Care and Sleep Medicine, Baylor College of Medicine, Houston, TX USA; 2 Institute for Clinical and Translational Research, Baylor College of Medicine, Houston, TX USA

**Keywords:** COVID-19, Critical care, Graduate medical education, Intensive care unit, Medical education, Pandemic, Procedures, Survey

## Abstract

**Background:**

The COVID-19 pandemic has changed the way medical education is delivered. The purpose of this study was to assess the impact of the COVID-19 pandemic on the education and procedural volume of critical care and pulmonary critical care fellows.

**Methods:**

We conducted a cross-sectional, internet-based, voluntary, anonymous, national survey of adult critical care fellows and academic attending physicians in critical care and pulmonary critical care fellowship programs in the United States between December 2020 and February 2021. Survey questions covered both didactic and non-didactic aspects of education and procedural volumes. Answers were ranked on a 5-point Likert scale. Survey responses were summarized by frequency with percentage. Differences between the responses of fellows and attendings were assessed with the Fisher’s exact or Chi-Square test, using Stata 16 software (StataCorp LLC, College Station, TX).

**Results:**

Seventy four individuals responded to the survey; the majority (70.3%) were male; less than one-third (28.4%) female. Respondents were evenly split among fellows (52.7%) and attendings (47.3%). 41.9% of survey respondents were from the authors’ home institution, with a response rate of 32.6%. Almost two-thirds (62.2%) reported that fellows spend more time in the ICU since the onset of the pandemic. The majority noted that fellows insert more central venous catheters (52.7%) and arterial lines (58.1%), but perform fewer bronchoscopies (59.5%). The impact on endotracheal intubations was mixed: almost half of respondents (45.9%) reported fewer intubations, about one-third (35.1%) more intubations. Almost all respondents (93.0%) described fewer workshops; and one-third (36.1%) fewer didactic lectures. The majority (71.2%) noted less time available for research and quality improvement projects; half (50.7%) noted less bedside teaching by faculty and more than one-third (37.0%) less fellow interaction with faculty. Almost one-half of respondents (45.2%) reported an increase in fellows’ weekly work hours.

**Conclusion:**

The pandemic has caused a decrease in scholarly and didactic activities of critical care and pulmonary critical care fellows. Fellows spend more time in ICU rotations, insert more central and arterial lines, but perform fewer intubations and bronchoscopies. This survey provides insights into changes that have occurred in the training of critical care and pulmonary critical care fellows since the onset of the COVID-19 pandemic.

**Supplementary Information:**

The online version contains supplementary material available at 10.1186/s12909-023-04358-2.

## Background

The Coronavirus-Disease-2019 (COVID-19) pandemic has changed the training and education of fellows in critical care (CCM) and pulmonary critical care medicine (PCCM), beyond well-described mental health challenges [1–3]. Social distancing, implemented to prevent disease spread, has necessitated a move to online or video lectures. Simulation workshops were cancelled or changed in format, to the potential detriment of learners [4, 5]. For example, 41% of pediatric gastroenterology fellows reported that their procedural experience was negatively impacted by the COVID-19 pandemic, and 30% felt it had a negative impact on their didactic learning [6]. In a national survey of cardiology fellows, a significant number were afraid of contracting COVID-19, exposing their friends and family to the disease, concerned about the potential inability to fulfill core training requirements, and feared limited job opportunities [7]. Twenty eight percent of pediatric anesthesiology fellows reported less clinical experience and education, and 22.7% were dissatisfied with their modified didactic experience [8]. Infection control policies that limited trainee access to COVID-19 positive patients and restricted family visitation may have also negatively affected the education of trainees [9, 10].

Becoming an intensivist requires both mastery of clinical knowledge and procedural competency; intensivists are expected to perform lifesaving procedures at the bedside of the critically ill patient [11]. A common concern during the early stages of the pandemic was that fellows may not have sufficient opportunities to obtain intubation and bronchoscopy skills. National societies published guidelines and recommendations that bronchoscopies should be avoided if possible and that the most experienced operator, typically an anesthesiologist, should intubate COVID-19 patients, because of the elevated risk of COVID-19 transmission during aerosolizing procedures [12–14]. Even before the pandemic, wide variation existed in whether CCM/PCCM fellows were the primary operators called upon to intubate intensive care unit (ICU) patients [15]. The well-documented lack of personal protective equipment (PPE) early in the pandemic, especially N95 masks, or Powered Air Purifying Respirators (PAPRs) used to protect proceduralists during intubations and bronchoscopies may have led to a reduction in the number of bronchoscopies performed in the ICU [13]. Additionally, patient volumes and case diversity were changed by the pandemic, with many fellows experiencing increased workload with potentially increased opportunities for procedures, but less breadth of diseases seen in the ICU.

Data about how the COVID-19 pandemic has affected the education and procedural volume of critical care trainees are lacking. To address this knowledge gap, we developed a survey of CCM/PCCM fellows and ICU attendings involved in teaching fellows.

## Methods

### Study design

We conducted a cross-sectional, web-based, voluntary, anonymous survey of fellows in United States (U.S.) CCM and PCCM training programs, as well as ICU attending physicians involved in fellowship education.

The Institutional Review Board (IRB) of Baylor College of Medicine reviewed and approved the study titled “Changes in clinical practice and critical care medicine fellowship education due to the COVID-19 pandemic; an anonymous online survey of graduate physicians and physicians enrolled in a critical care medicine or combined pulmonary/critical care medicine fellowship program” on 12/08/2020, IRB # H-48817, with a waiver of signed consent. The participants’ decision to complete the survey after reviewing the study information was considered their consent to participate. The study was conducted in accordance with the IRB’s ethical standards on human experimentation and with the Helsinki Declaration of 1975.

### Survey development

The authors developed the survey by identifying common procedures performed by fellows in the ICU: central line insertion, arterial line insertion, endotracheal intubation, bronchoscopy, chest tube placement, percutaneous tracheostomy, thoracentesis, paracentesis, pulmonary artery catheter insertion, and point-of-care ultrasound. We identified important educational aspects of CCM/PCCM fellowship programs based on the ACGME Common Program Requirements for Critical Care Medicine and the authors’ experience as fellowship leaders [11]: frequency and format of didactic lectures and simulation workshops; bedside teaching by faculty; fellow independence; diversity of cases; average weekly work hours; time spent in ICU rotations; time available for research and quality improvement (QI). Based on changes we noted in our institutions after the onset of the pandemic, we identified additional elements affecting the fellowship experience, such as time fellows spend interacting with families and how often fellows perform a physical exam in COVID-19 patients. We arrived at the final survey questions by consensus after several rounds of review. We pilot-tested the survey questions for clarity and response time with trainees and integrated their feedback. The survey consisted of 10 demographic questions and 4 questions about changes in procedural volume and fellowship education.

Participants were asked to compare their current pandemic experience to before the pandemic on a 5-point Likert scale.

While our study is the first instance of usage of these specific survey questions, the questions are considered to possess content validity since they addressed the most common ICU procedures, including ACGME-required procedures for critical care board certification, and typical and universal educational aspects of U.S. critical care fellowship programs. Internal reliability of the survey is supported by respondents providing concordant answers to related questions about various educational formats (i.e. didactic lectures, workshops, bedside teaching by faculty).

### Survey distribution

The study was conducted between December 2020 and February 2021. We distributed the survey to all ICU attendings and fellows at Baylor College of Medicine by direct email invitation. We also sent an email invitation to all CCM and PCCM fellowship program directors and coordinators nationwide; their email addresses are available through the Fellowship and Residency Electronic Interactive Database (FREIDA™) [16]. We requested that they forward the survey to their trainees and teaching faculty. Several reminder emails were sent. We explored other options to distribute the survey to the target audience, but no mechanism exists to distribute a survey to all U.S. CCM/PCCM fellows and ICU teaching faculty.

### Data analysis

Study data were collected and managed using REDCap electronic data capture tools hosted at Baylor College of Medicine [17, 18]. REDCap (Research Electronic Data Capture) is a secure, web-based software platform designed to support data capture for research studies, providing 1) an intuitive interface for validated data capture; 2) audit trails for tracking data manipulation and export procedures; 3) automated export procedures for seamless data downloads to common statistical packages; and 4) procedures for data integration and interoperability with external sources.

Statistical analysis was performed by using Stata 16 software (StataCorp LLC, College Station, TX). Categorical variables were summarized by using percentages rounded to the first decimal. Responses were stratified by attendings and fellows and compared using the Fisher’s exact or Chi-square test. A *P*-value of < 0.05 was considered statistically significant.

## Results

### Characteristics of study participants

The demographic characteristics of the study participants are shown in Table 1. Among 74 respondents who filled out the complete survey, 47.3% were ICU attending physicians who supervised fellows, and 52.7% were fellows in critical care training programs (64.1% PCCM; 30.8% CCM; 5.1% neurocritical care). The majority (70.3%) of respondents were male. There were no statistically significant differences between attendings and fellows regarding the U.S. region they practiced in (*p* = 0.118), their clinical practice setting (*p* = 0.797), or the number of clinical fellows in the program (*p* = 0.174). 41.9% of all survey respondents (38.5% of all fellows and 45.7% of all attendings) were from Baylor College of Medicine, the authors’ home institution, and their overall response rate was 32.6% (46.9% for fellows, 25.4% for attendings).

**Table 1:** Characteristics of study participants

**Characteristic**		**All participants % (number)**	**Fellows**	**Attendings**
**Role**		100% *N* = 74	52.7% *N* = 39	47.3% *N* = 35
**Gender**	Female	28.4% (21)	35.9% (14)	20.0% (7)
	Male	70.3% (52)	61.5% (24)	80.0% (28)
	Prefer not to disclose	1.4% (1)	2.6% (1)	0.0% (0)
**US Region**	East North Central (IL, IN, MI, OH, WI)	1.4% (1)	0.0% (0)	2.9% (1)
	East South Central (AL, KY, MS, TN)	6.8% (5)	2.6% (1)	11.4% (4)
	Mid-Atlantic (NJ, NY, PA)	10.8% (8)	17.9% (7)	2.9% (1)
	Mountain (AZ, CO, ID, MT, NM, NV, UT, WY)	2.7% (2)	5.1% (2)	0.00% (0)
	New England (CT, MA, ME, NH, RI, VT)	6.8% (5)	2.6% (1)	11.4% (4)
	Pacific (AK, CA, HI, OR, WA)	5.4% (4)	5.1% (2)	5.7% (2)
	South Atlantic (DC, DE, FL, GA, MD, NC, SC, VA, WV)	12.2% (9)	12.8% (5)	11.4% (4)
	West North Central (IA, KS, MN, MO, ND, NE, SD)	0.0% (0)	0.0% (0)	0.0% (0)
	West South Central (AR, LA, OK, TX)	54.1% (40)	53.8% (21)	54.3% (19)
**Clinical Practice Setting**	Academic center	93.2% (69)	94.9% (37)	91.4% (32)
	Community based, academic affiliation	5.41% (4)	5.1% (2)	5.7% (2)
	Veterans Administration Health Care	1.35% (1)	0.0% (0)	2.9% (1)
**Number of Clinical Fellows in program**	< 5 fellows	12.2% (9)	5.1% (2)	20.0% (7)
	5–10 fellows	17.6% (13)	15.4% (6)	20.0% (7)
	11–15 fellows	37.8% (28)	48.7% (19)	25.7% (9)
	16–20 fellows	20.3% (15)	20.5% (8)	20.0% (7)
	> 20 fellows	12.2% (9)	10.3% (4)	14.3% (5)
**Fellowship year**	1^st^ year fellow		33.3% (13)	
	2^nd^ year fellow		41.0% (16)	
	3^rd^ year fellow		25.6% (10)	
**Training program**	Pulmonary and critical care medicine		64.1% (25)	
	Critical care medicine		30.8% (12)	
	Neurocritical care medicine		5.1% (2)	
**Years since graduation from fellowship**	< 5 years ago			17.1% (6)
	5–10 years ago			25.7% (9)
	11–20 years ago			25.7% (9)
	> 20 years ago			28.6% (10)
	No critical care subspecialty training			2.9% (1)

### Changes in fellows’ procedural volume

**Fig. 1:**
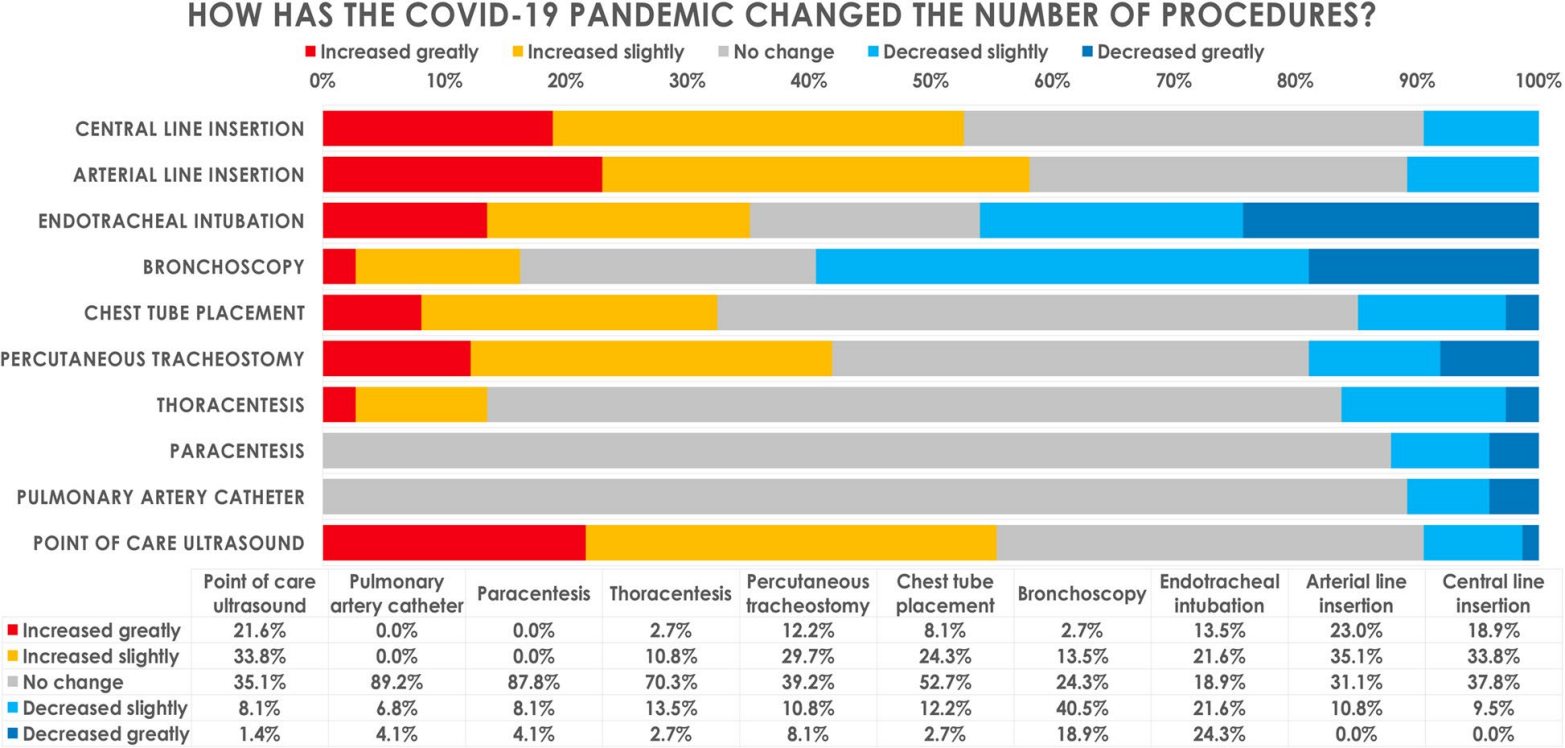
How has the COVID-19 pandemic changed the number of ICU procedures performed by fellows?

Changes in the numbers of critical care procedures performed by fellows during the pandemic compared to before the pandemic are shown in Fig. 1. Bronchoscopies were the procedures most negatively impacted by the pandemic. The majority (59.5%) reported a decrease in the number of bronchoscopies performed by fellows; far fewer reported no change (24.3%) or an increase (16.2%). Almost half of respondents (45.9%) reported a decrease in endotracheal intubations, whereas one-third (35.1%) noted an increase. A slight majority of respondents (52.7%) reported no change in the number of chest tube insertions, whereas one-third (32.4%) reported an increase. The majority reported that fellows placed more central lines (52.7%) and more arterial lines (58.1%), and that fellows utilized more point-of-care ultrasound (55.4%). A significant proportion of respondents (41.9%) noted that fellows performed more percutaneous tracheostomies during the pandemic. Procedures whose volume remained unaffected by the pandemic included thoracentesis for which almost three-quarters of respondents (70.3%) reported no change; almost all respondents noted no change in the number of paracentesis (87.8%) and pulmonary artery catheter insertions (89.2%).

### Changes in fellows’ general education

**Fig. 2:**
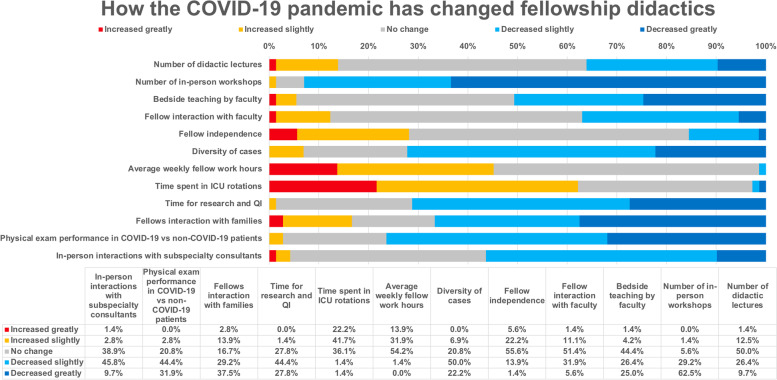
How the COVID-19 pandemic has changed fellowship didactics

The effects of the pandemic on fellows’ general education are shown in Fig. 2. More than one-third (36.1%) of respondents reported a decrease in didactic lectures, half (50.0%) no change, and a minority (13.9%) an increase. Almost all respondents noted that the number of in-person procedure and simulation workshops had declined (93.0%). The learning that occurs in direct fellow and faculty interaction has also been affected by the pandemic: half of the respondents (50.7%) reported less bedside teaching by faculty. Slightly more than one-third of participants (37.0%) noted a decrease in fellow interaction with faculty, whereas half (50.7%) reported no change. Almost three-quarters of respondents (72.2%), described a decrease in the case diversity in the ICU. A slight majority (53.4%) reported no change in fellows’ average weekly work hours during the pandemic, but 45.2% reported an increase. A majority (62.2%) reported that fellows spend more time in ICU rotations; one-third (35.1%) noted no change. Almost three-quarters of respondents (71.2%) described fellows having less time for research and QI efforts.

Two-thirds of respondents (66.7%) felt that fellows interacted less with families since the pandemic. Three-quarters (76.4%) noted a decrease in how often fellows examined COVID-19 patients compared to non-COVID-19 patients. A large majority (85.3%) of participants noted that many subspecialists asked to consult on COVID-19 patients were less likely to enter a patient room and personally examine the patient compared to non-COVID-19 patients. In-person fellow interaction with subspecialty consultants has also decreased due to the COVID-19 pandemic, according to a majority (56.3%) of respondents.

### Changes in didactic fellowship conferences

**Fig. 3:**
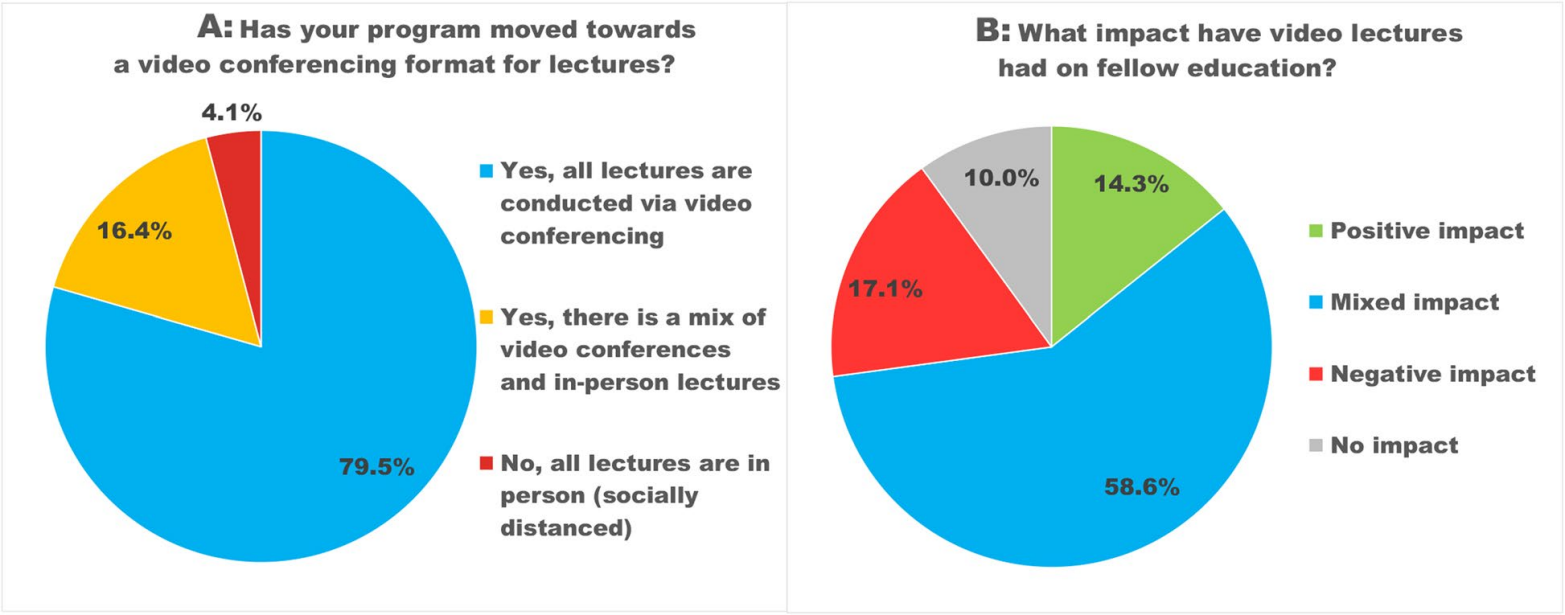
Lecture format and impact of video lectures **A** Has your program moved towards a video conferencing format for lectures? *Legend*: 79.5% reported that all lectures are conducted via video conferencing; 16.4% had a mix of video conferences and in-person lectures, and 4.1% conducted all lectures in person (socially distanced) **B** What impact have video lectures had on fellow education? Legend: 14.3% reported a positive impact, 58.6% a mixed impact, 17.1% a negative impact, and 10% no impact of the video lecture format on fellow education

Figure 3A depicts how the COVID-19 pandemic has changed the format of fellowship conferences (i.e., didactic sessions for the fellows). A large majority (79%) of participants reported that all lectures are conducted via video-conferencing, and most (58.6%) felt this had a mixed impact. **(**Fig. 3B**)** Common themes in free-text comments about the educational consequences of video-conferencing lectures include that better lecture attendance, convenience, and the ease of inviting outside speakers count among the advantages of video lectures. Many were concerned about decreased opportunities for fellows to interact with each other and with faculty. Fellows appreciated the ability to watch recorded lectures that they had missed, or re-watch lectures, but reported decreased engagement and frequent distractions during video lectures. Attendings noted less discussion, fewer questions and difficulty assessing learner understanding. (Table 2).

**Table 2:** Illustrative quotes from survey responses to the open-ended question: What impact has the move towards a video conferencing format for lectures had on fellow education?

Survey responses by fellows	
Positive	• “More compliance due to lack of need to travel between pavilions.” • “Able to attend more lectures online despite being in different pavilions and able to get speakers outside of institution more easily.”
Mixed	• “More lectures, but less interaction and difficult to engage.” • “Many lectures are recorded, which gives the option to view at a later time if I would have otherwise missed the lecture if only offered live. However, it is also hard to have uninterrupted time without any distractions for these video lectures so I frequently find myself "double tasking" during them, which does effect the amount of knowledge I gain.” • “Easier and more comfortable to receive the lectures however we miss getting to know our co fellows, interacting.” • “Exposure to a novel disease is good but everything else has been suboptimal.” • “It's a lot more difficult to stay engaged by video but my attendance has improved since it's all by zoom.” • “Easier to attend, but also since I don't leave the work site the time is not as protected.” • “Less engaging, but the opportunity to go over the lecture again is a positive thing.”
Negative	• “Lack of hands on didactics, procedures.” • “There is less interaction.” • “Less accountability in teaching and interaction.” • “Overall decrease in didactics and lectures; significantly less experience in pulmonary compared to critical care.” • “Less effective.” • “Lack of informal discussion surrounding lectures.” • “Unknown whether people are actually in the lecture vs just logged in the computer.” • “Lost in person interaction and used to have richer discussions and better questions.”
Survey responses by attendings	
Positive	• “Improved attendance at conference.” • “Easier to participate; also easier to get speakers from other centers as there is no need for travel.” • “Increased compliance with lecture schedule, increased attendance by fellows and attendings.” • “Better participation.” • “Increased attendance.”
Mixed	• “Some improvement in accessibility at the cost of decrease in interaction and limitations on format imposed by the video medium.” • “Positive: easier attendance. Negative: camaraderie is less I think.” • “Good attendance; less in-person interactions.” • “Less interaction but more likely to be available and present.” • “I am unsure what impact it has had on the fellows. I imagine a negative impact due to the video conference format and the reduced ability to interact as a group. The number of conferences remains unchanged.” • “Great for ease of access, but has decreased the interaction between faculty and fellows.”
Negative	• “Less in-person contact makes asking questions and assessing understanding difficult.” • “Less feedback and open forum during video conference.” • “Less discussion.” • “Interaction is limited compared to being there.” • “Often have technical problems.” • “Less Q&A interactions.” • “Decreased interaction with colleagues and faculty.”

When asked about other ways in which fellowship education has changed during the COVID-19 pandemic, both fellows and attendings voiced concerns about less procedural training and fewer hands-on workshops, less procedures especially airway management, less time for scholarly activities due to an increased number of ICU rotations, increased burnout and moral distress, significantly less social interaction among each other and with faculty, and difficulty finding a post-graduation job (Table 3).

**Table 3:** Illustrative quotes from survey respondents to the open-ended question: In what other ways has fellowship education changed during the COVID-19 pandemic?

**Practical skills**
• “Procedural training has been affected.” (fellow) • “Less hands-on workshop time was definitely detrimental for our incoming fellows.” (fellow) • “Worrisome for less rotations, less intubations.” (fellow) • “Rotations that were not supposed to be ICU turned into ICU rotations. This is one extra ICU month for someone that has at least 9 months of ICU / year. Expectations and evaluations regarding practical skills were the same as prior to the pandemic. However, we had less opportunity to do so due to the pandemic and were harshly evaluated. No procedure workshop or ultrasound bootcamp was done. However, the expectation that fellows needed to be proficient was still there. If the program didn't provide the proper educational resources early in the year it is unreasonable to expect proficiency without teaching.” (fellow) • “Less pulmonary rotations including procedures like EBUS.” (fellow) • “Increased POCUS learning and ARDS knowledge.” (fellow)
**General education**
• “Lack of case variety.” (fellow) • “Less time for spontaneous teaching on rounds.” (fellow) • “All education is now online.” (fellow) • “Decreased informal gatherings to discuss cases and literature.” (attending) • “Reduction of direct patient contact is an important issue.” (attending) • “Decreased exposure to non-COVID and non-ICU topics of importance. Disruption of didactic curriculum.” (attending) • “Outpatient clinics have evolved into more telemedicine which is as many things in life, good and bad.” (attending) • “There is less diversity of cases seen by the fellows this however has been associated with increased exposure to ARDS and its complications.” (attending) • “Less diversity of cases.” (attending) • “Better lectures from outside well renowned lecturers.” (attending) • “The education has improved.” (attending)
**Mental health**
• “Much less social interaction. It took almost 6 months to meet 1st year fellows which had a significant impact on morale and therefore motivation.” (fellow) • “Less interaction with peers has led to a loss of perspective on day-to-day life in the ICU.” (fellow) • “Increased burnout/moral distress given PCCM fellows and faculty carry brunt of COVID ICU care.” (fellow) • “Less interaction between faculty and fellows.” (fellow) • “Impersonal. Less interaction with family.” (fellow) • “Less time off, less conference, less exposure to outside networking. Job hunting mostly online. Pandemic was terrible.” (fellow) • “Stress level, increased mortality, moral distress particularly around families not able to visit.” (attending) • “Less camaraderie, more "in the dark" as to what is happening so feels less like a family.” (attending) • “Less interaction.” (attending)
**Scholarship**
• “Less elective/research and pulmonary rotations.” (fellow) • “Decreased research opportunities.” (fellow) • “Less focus on research and teaching residents/med students.” (fellow) • “Less opportunity for scholarship at this point.” (attending) • “Less time for research.” (attending)

### Comparison of attending and fellow responses

We compared the responses given by fellows with those by attending physicians, to assess whether their answers differed. Significantly more fellows than attendings reported that fellows performed more percutaneous tracheostomies (*p* = 0.036), fewer intubations (*p* = 0.035) and spent more time in ICU rotations (*p* = 0.046), compared to before the pandemic.

## Discussion

### Principal findings

In this survey, critical care fellows and attending intensivists involved in fellow education self-reported how the COVID-19 pandemic has affected various aspects of fellow education in the ICU, including didactic and nondidactic teaching, relative time spent on various activities, procedural volumes, interaction with patients and families, and scholarship.

The number of fellows and attendings who responded to the survey was similar, which provides a good balance of perspectives. The gender distribution of the survey participants, with 70.3% men and 28.4% women, reflects the composition of the U.S. ICU physician workforce where only 26.8% of practicing intensivists are women [19]. Women were better represented among fellows than attendings (35.9% compared to 20%), similar to the gender distribution of PCCM/CCM fellows described in the literature [20].

The survey responses demonstrate that fellows and attendings perceive that the relative allocation of the activities that are required components of CCM and PCCM fellowship training in the U.S. have been significantly changed by the pandemic. The majority of respondents noted that fellows had more ICU rotations than before the pandemic, which is not surprising, but this implies that fellows had less time for non-ICU clinical and non-clinical scholarly activities. Indeed, a large majority reported that fellows had less time available for research and quality improvement projects. Almost half the participants reported an increase in the total weekly work hours for fellows, compared to before the pandemic. The total number of typical ICU procedures performed by fellows was altered by the pandemic as well: Fellows inserted more central venous and arterial lines, but performed fewer bronchoscopies and endotracheal intubations. Both didactic and nondidactic teaching of fellows has been negatively affected by the pandemic: Almost all respondents described that fellows participated in fewer workshops, which are essential for the acquisition of procedural and other practical skills (e.g. familiarity with ventilators, mechanical circulatory support devices, communication skills). About half of the respondents noted less bedside teaching by faculty, and one-third reported a reduction in the total number of didactic lectures. More than one-third of participants noted that fellows interacted less with teaching faculty. This indicates that CCM/PCCM fellows received less formal and informal education compared to before the pandemic.

### Implications and relation to other studies

#### Pandemic Effects on ICU Procedures

The decreased number of airway procedures may have long-term negative effects on ICU patient care, since procedural skills are crucial for the practicing intensivist. The reduction in the number of bronchoscopies is concordant with national guidelines and likely related to concerns about COVID-19 transmission during this aerosolizing procedure [13, 21, 22]. A survey of interventional pulmonology fellows similarly noted a decline in bronchoscopies since the pandemic [23]. The reduction in the number of intubations by fellows could be explained by hospital policies early in the pandemic assigning anesthesiologists to intubate all COVID-19 patients, or attempts by faculty to protect trainees from COVID-19 exposure. In other hospitals, the large number of ICU patients with severe acute respiratory distress syndrome (ARDS) from COVID-19 may have given fellows more opportunities to perform intubations. While three-year PCCM fellows have time to catch up on procedural skills, this may not be true for one- or two-year critical care fellows. The increase in central and arterial line insertions by fellows likely reflects the absolute increase in the number of ICU patients due to the pandemic. Fellows inserting more chest tubes may be related to the high frequency of barotrauma leading to pneumothorax observed in COVID-19 patients, or the overall high number of patients with ARDS. The large number of COVID-19 ARDS survivors requiring prolonged mechanical ventilation might explain why fellows had more opportunities to perform percutaneous tracheostomies. The decline in hands-on workshops noted by almost 90% of survey participants may negatively affect the procedural competency of CCM and PCCM fellows, in particular airway management skills; traditionally, programs have utilized simulation training to help fellows acquire these skills [24]. In contrast to our study, surgical residents reported that only 53% of simulation training was suspended by the pandemic [25]. Fellows reported more frequently than attendings that they performed fewer intubations and spent more time in ICU rotations since the pandemic. Attendings may be less aware of fellow concerns about inadequate training in airway management skills during the pandemic, or less aware of how many procedures fellows perform.

In a recently published survey of PCCM program directors (PDs) from 2020, 77.6% of the PDs reported that PCCM fellows spent more time in the ICU than originally scheduled and performed fewer elective outpatient bronchoscopies [26]. Similar to our findings, this study reported an increase in central and arterial line insertions and a reduction in both ICU and elective outpatient bronchoscopies, but in contrast to our study, the PDs noted that fellows performed fewer intubations and percutaneous tracheostomies. Our survey was directed to a different audience (fellows and teaching faculty rather than PDs; both CCM and PCCM fellows instead of exclusively PCCM fellows), and we focused exclusively on ICU procedures and critical care education.

#### Pandemic effects on fellows’ learning experience

Reported changes in the learning experience of fellows in the ICU since the pandemic include fewer didactic lectures, longer work hours in the ICU, less time for scholarly activities (research and QI projects), less bedside teaching, and fewer interactions with critical care and subspecialty consultants, which may affect fellows’ knowledge acquisition in areas such as infectious disease or cardiology. Others have similarly reported that fellows have been reassigned to clinical duties from their research rotations [14]. Fellowship lectures have moved online, though it is surprising that only 80% experienced this. Training programs in cardiology, pediatric anesthesiology and ophthalmology have reported a comparable shift to video conferences, in response to social distancing guidelines and prohibition of in-person gatherings [7, 8, 14, 27].

Participants reported that fellows perform a physical exam less frequently in COVID-19 patients, compared to non-COVID-19 patients, which may negatively impact trainees’ clinical skills. Potential reasons include the lack of PPE early in the pandemic, hospital policies limiting the number of staff interacting with COVID-19 patients, and fear and anxiety about contracting COVID-19 or carrying the infection home to loved ones, leading to minimization of contact with COVID-19 patients [28].

About two thirds of fellows and attendings indicated that fellows interact less with families since the pandemic; contact with families occurs through phone calls, likely due to family visitation restrictions. The negative impact of visitation restrictions on family members and patients has been studied, but the consequences for fellow education have not been explored [29–31]. The implementation of visitation restrictions for COVID-19 patients has been linked to negative mental health outcomes such as depression, anxiety and peritraumatic dissociation in ICU physicians [32]. Some of the free-text survey responses suggest trainee isolation and struggles and portend a growing mental health and burnout crisis.

### Limitations and strengths

Our study has several limitations. The number of participants was lower than expected, and a significant percentage of respondents (41.9%) were from the authors’ home institution. The response rate for participants from Baylor College of Medicine was 32.6%, which is a good response rate for this type of survey. We cannot calculate an overall survey response rate, since it is unknown how many of the program directors and program coordinators who received the survey link forwarded the survey to their fellows and teaching faculty (likely only a minority), or what the total number of U.S. ICU attendings is. Error may have been introduced by participants misremembering the past. We were unable to examine objective procedure logs. Respondents were mainly first- and second-year fellows, partly because 30% of participants were CCM fellows in one- or two-year training programs, whereas a PCCM fellowship lasts 3 years. First-year fellows were not excluded since they had pre-pandemic ICU experiences during residency training as a point of comparison. Potential differences between fellows depending on their year of training may have skewed answers in research-heavy programs, though the survey responses indicate that many fellows were pulled from research to clinical rotations in the first year of the pandemic, making such a bias less likely.

The survey was conducted early in the pandemic (December 2020 through February 2021), and programs have iteratively changed since then, in response to vaccine availability and changing CDC guidance, so results might have been different later in the pandemic.

Nevertheless, the study demonstrates how the cohort of fellows training during the first year of the pandemic has been affected. This is the first study assessing how ICU fellows and attendings perceive in what ways the COVID-19 pandemic has changed fellowship training both from a didactic and procedural perspective. The survey results and free text comments can be useful for program directors considering which changes should be retained (e.g. recording lectures and making them available for post-call fellows), what the deficiencies in fellow education are and how they can be remediated. The study points to an increased need for more training in airway procedures. The free text comments add to the study because they allow insight into what the fellows appreciated about the video conferencing of lectures, but also missed most – the personal interaction with other fellows and attendings. Multiple comments address the negative mental health consequences of limited personal interaction between fellows and attendings, and patients and families as well, and increased burnout and moral distress caused by the pandemic.

## Conclusion

The pandemic has led to a decrease in scholarly and didactic activities of CCM and PCCM fellows, while fellows spend more time in ICU rotations. The amount of ICU procedures performed by fellows has also changed—fellows place more central and arterial lines, but perform fewer intubations and bronchoscopies. Capturing the perspective of fellows and teaching faculty can help critical care fellowship programs identify areas of weakness, particularly in procedural skills, to develop strategies to mitigate the adverse effects of the COVID-19 pandemic on fellowship training in the ICU. Educators need to target procedural skills and emphasize simulation training and one-on-one learning from attending role models, learn to utilize technology for learning, attempt to compensate for the loss of research time, and address the mental health needs of trainees. The survey results underline the importance of in-person interaction between fellows and attendings that cannot be supplanted by virtual connections.

## Key Points

### Question

How has the COVID-19 pandemic changed the procedural volume and education of fellows in the ICU?

### Findings

In a cross-sectional survey of ICU fellows and attendings, the participants reported that the COVID-19 pandemic has led to a decrease in fellows’ scholarly and didactic activities. Fellows spend more time in ICU rotations, insert more central and arterial lines, but perform fewer intubations and bronchoscopies. They interact less with faculty and consultants.

### Meaning

Intensivists who supervise fellows can use the survey results to target specific areas in fellowship training that have been negatively affected by the COVID-19 pandemic, particularly potential deficiencies in airway management skills.

## Brief Summary

In a cross-sectional survey of ICU fellows and attendings, the participants reported that the COVID-19 pandemic has led to a decrease in fellows’ scholarly and didactic activities. Fellows insert more central and arterial lines, but perform fewer intubations and bronchoscopies.

## Supplementary Information

**Additional file 1.**

## Data Availability

The datasets generated and analyzed during the current study are not publicly available, due to IRB—mandated human subject protection, but are available from the corresponding author on reasonable request.

## Abbreviations

ARDS: Acute respiratory distress syndrome
CCM: Critical Care Medicine
COVID-19: Coronavirus disease 2019
FREIDA: Fellowship and Residency Electronic Interactive Database
ICU: Intensive Care Unit
IRB: Institutional Review Board
PAPR: Powered Air Purifying Respirators
PCCM: Pulmonary and Critical Care Medicine
PD: Program Director
PPE: Personal protective equipment
QI: Quality Improvement
REDCap: Research Electronic Data Capture
TX: Texas
U.S.: United States
