# Supplementary material for: Impact of the COVID-19 pandemic on the education and procedural volume of fellows in critical care medicine – a cross-sectional survey
Source: BMC Med Educ. 2023 May 24;23:371. doi: 10.1186/s12909-023-04358-2 (PMC10206548; doi:10.1186/s12909-023-04358-2)
Supplement: Supplementary file 1 — Additional file 1. [file 12909_2023_4358_MOESM1_ESM.pdf]

1. Where do you practice critical care medicine (U.S. region)?	◯ New England (CT, MA, ME, NH, RI, VT)<br>◯ Mid-Atlantic (NJ, NY, PA)<br>◯ East North Central (IL, IN, MI, OH, WI)<br>◯ West North Central (IA, KS, MN, MO, ND, NE, SD)<br>◯ South Atlantic (DC, DE, FL, GA, MD, NC, SC, VA, WV)<br>◯ East South Central (AL, KY, MS, TN)<br>◯ West South Central (AR, LA, OK, TX)<br>◯ Mountain (AZ, CO, ID, MT, NM, NV, UT, WY)<br>◯ Pacific (AK, CA, HI, OR, WA)
2. What best describes your clinical practice setting?	◯ Academic center<br>◯ Community based, academic affiliated<br>◯ Community based<br>◯ Private practice<br>◯ Other - please specify
3. What is your role in the ICU?	◯ Attending physician<br>◯ Fellow
i. How many years ago did you graduate from critical care or pulmonary/critical care fellowship?	◯ < 5 years ago<br>◯ 5-10 years ago<br>◯ 11-20 years ago<br>◯ > 20 years ago<br>◯ I have not received fellowship training in critical care or pulmonary/critical care medicine
ii. Do you actively practice medicine in the intensive care unit?	◯ Yes<br>◯ No
iii. Do you supervise fellows in the ICU?	◯ Yes<br>◯ No
i. In what specialty is your training program?	◯ Pulmonary-Critical Care Medicine<br>◯ Critical Care Medicine<br>◯ Pulmonary Medicine<br>◯ Emergency medicine - critical care medicine<br>◯ Anesthesia critical care<br>◯ Neurocritical care<br>◯ Surgical critical care<br>◯ Other - please specify
	________________________________
ii. What year of fellowship are you in?	◯ First year<br>◯ Second year<br>◯ Third year<br>◯ Fourth year
iii, How many clinical fellows are in your program (all years)?	◯ < 5 fellows<br>◯ 5-10 fellows<br>◯ 11-15 fellows<br>◯ 16-20 fellows<br>◯ > 20 fellows<br>◯ Don't know
4. What is your gender?	◯ Female<br>◯ Male<br>◯ Prefer not to disclose

5. How has the COVID-19 pandemic impacted the number of procedures (listed below) performed by fellows in your program? The number of procedures has:

	Increased greatly	Increased slightly	No change	Decreased slightly	Decreased greatly
Central line insertion	◯	◯	◯	◯	◯
Arterial line insertion	◯	◯	◯	◯	◯
Endotracheal intubation	◯	◯	◯	◯	◯
Bronchoscopy	◯	◯	◯	◯	◯
Chest tube placement	◯	◯	◯	◯	◯
Percutaneous tracheostomy	◯	◯	◯	◯	◯
Thoracentesis	◯	◯	◯	◯	◯
Paracentesis	◯	◯	◯	◯	◯
Pulmonary artery catheter placement (Swan-Ganz catheter)	◯	◯	◯	◯	◯
Point of care ultrasound	◯	◯	◯	◯	◯

6. In what ways has the pandemic changed fellowship education (compare education during the pandemic to education before the pandemic)?

	Increased greatly	Increased slightly	No change	Decreased slightly	Decreased greatly	Don't know
Number of didactic lectures	◯	◯	◯	◯	◯	◯
Number of in-person, hands-on workshops (e.g. procedural skills workshops, simulation training)	◯	◯	◯	◯	◯	◯
Bedside teaching by faculty	◯	◯	◯	◯	◯	◯
Fellow interaction with faculty	◯	◯	◯	◯	◯	◯
Fellow independence	◯	◯	◯	◯	◯	◯
Diversity of cases seen during the pandemic, compared to before the pandemic	◯	◯	◯	◯	◯	◯
Time fellows spend interacting with families	◯	◯	◯	◯	◯	◯
Frequency of fellows performing a physical exam (in COVID-19 compared to non-COVID-19 patients)	◯	◯	◯	◯	◯	◯
Average weekly work hours for fellows	◯	◯	◯	◯	◯	◯
Time spent in ICU rotations	◯	◯	◯	◯	◯	◯
Time available for research and quality improvement	◯	◯	◯	◯	◯	◯
In-person interactions with subspecialty consultants	◯	◯	◯	◯	◯	◯
Frequency of subspecialty consultants performing a physical exam in COVID-19 patients compared to non-COVID-19 patients	◯	◯	◯	◯	◯	◯

7. Has your program moved towards a video conferencing format for lectures?	◯ Yes, all lectures are conducted via video conferencing<br>◯ Yes, there is a mix of video conferences and in-person lectures<br>◯ No, all lectures are in person (socially distanced)<br>◯ No, we stopped using lectures as an educational tool<br>◯ Other - please specify
	________________________________
i. If yes: What impact has this had on fellow education?	◯ Positive impact - please specify (optional)<br>◯ Mixed impact - please specify (optional)<br>◯ Negative impact - please specify (optional)<br>◯ No impact
	________________________________
8, In what other ways has fellowship education changed during the COVID-19 pandemic?	________________________________
